# Differential effects of smoking on lung cancer mortality before and after household stove improvement in Xuanwei, China

**DOI:** 10.1038/sj.bjc.6605791

**Published:** 2010-07-20

**Authors:** K-M Lee, R S Chapman, M Shen, J H Lubin, D T Silverman, X He, H D Hosgood, B E Chen, P Rajaraman, N E Caporaso, J F Fraumeni, A Blair, Q Lan

**Affiliations:** 1 Division of Cancer Epidemiology and Genetics, National Cancer Institute, Bethesda, MD, USA; 2 College of Public Health Sciences, Chulalongkorn University, Bangkok, Thailand; 3 Institute of Environmental Health and Engineering, Chinese Academy of Preventive Medicine, Beijing, China; 4 Department of Epidemiology and Public Health, Yale School of Medicine, New Haven, CT, USA; 5 Department of Community Health and Epidemiology, Queens University, Kingston, Ontario, Canada

**Keywords:** smoky coal, smoking, lung cancer, mortality, Xuanwei cohort, time-dependent variable

## Abstract

**Background:**

In Xuanwei County, Yunnan Province, China, lung cancer mortality rates in both males and females are among the highest in China.

**Methods:**

We evaluated differential effects of smoking on lung cancer mortality before and after household stove improvement with chimney to reduce exposure to smoky coal emissions in the unique cohort in Xuanwei, China. Effects of independent variables on lung cancer mortality were measured as hazard ratios and 95% confidence intervals using a multivariable Cox regression model that included separate time-dependent variables for smoking duration (years) before and after stove improvement.

**Results and conclusion:**

We found that the effect of smoking on lung cancer risk becomes considerably stronger after chimney installation and consequent reduction of indoor coal smoke exposure.

In Xuanwei County, Yunnan Province, China, lung cancer mortality rates in both males and females are among the highest in China (Mumford *et al*, 1987), and attributed to polycyclic aromatic hydrocarbon (PAH) and nitrogen heterocyclic compounds contained in smoke from coal combustion (Mumford *et al*, 1987, 1990).

In a retrospective cohort study conducted in 1992, we noted a significantly decreased risk of incident lung cancer after household stove improvement (Lan *et al*, 2002). Interestingly, we observed only a weak effect of smoking on lung cancer, which was consistent with previous case–control studies conducted in Xuanwei (He *et al*, 1991; Lan *et al*, 2000). Given that cigarette smoking has been strongly related to high incidence or mortality of lung cancer (Doll and Peto, 1981), we suspected that high exposure to indoor airborne carcinogens from coal smoke might partly explain the weak effect of smoking in this cohort.

In this study with extended follow-up, we compared the separate effects of smoking on lung cancer mortality before and after stove improvement with chimney.

## Materials and methods

Details of this cohort study have been described previously (Lan *et al*, 2002). In 1992, local administrative records were searched to identify all farmers born between 1917 and 1951 who were living in Xuanwei's communes as of 1 January 1976. Follow-ups of the study participants (*n*=42 422) for vital status and cause of death were carried out in 1992 and 1996. As this paper focuses on the effect of smoking before and after stove improvements, women subjects (*n*=20 721), among which only <1% were ever-smokers, were excluded. Out of 21 701 male subjects, a total of 12 529 (52.2%) installed chimneys, and a total of 6580 changed to portable stoves. For a direct comparison of the results of the Lan *et al* (2002), change to portable stoves was not considered in this study.

### Statistical analysis

To evaluate effects of smoking, the Cox models previously developed for males were slightly modified to estimate hazard ratios (HRs) and 95% confidence intervals (CIs) using the SAS PHREG procedure (SAS 9.1. 3, Cary, NC, USA). The time variable (time axis) was age, starting with age as of 1 January 1976 and ending with age at exit from the follow-up. The outcome event was death from lung cancer. Time-dependent explanatory variables included stove improvement (switching from 0 to 1 at the age of improvement), smoky coal use (tonnage per year), smoking duration (years), and cooking (ever *vs* never). Non-time-dependent variables included in the models were the same as Lan *et al* (2002). To adjust for potential birth cohort effects, models were stratified into seven birth cohorts: 1917–1921, 1922–1926, 1927–1931, 1932–1936, 1937–1941, 1942–1946, and 1947–1951.

We further modified the Cox model to estimate the effects of smoking before and after stove improvement, with two separate time-dependent variables of smoking replacing the variable for overall effect of smoking. Then, to estimate the magnitude and statistical significance of interaction between smoking and stove improvement, interaction terms of time-dependent smoking variables with the time-dependent stove improvement variable were added to the Cox models.

As smoking type may have changed over the study period (e.g., from pipe to cigarettes) and the effect on lung cancer risk differs by smoking type (Benhamou *et al*, 1986), we conducted smoking type-specific analyses. Risks for cumulative exposure (pack-years) to specific smoking types (cigarette: filtered/unfiltered, Chinese long-stem pipe, and water pipe) were characterised and compared in separate reduced data sets for each type, which included exclusive lifetime smokers of each type and non-smokers.

## Results

A total of 12 529 subjects (55.2%) changed permanently to stoves with chimney and 9172 (44.8%) did not (the reference group) (Table 1). As previously reported, changing to stoves with chimneys was significantly associated with a reduced risk of lung cancer mortality (HR=0.70, 95% CI=0.62–0.80) (data not shown).

After stove improvement, the effect of smoking duration (per year) on lung cancer mortality significantly increased (HR_interaction_=1.015, *P*_interaction_=0.002) (Table 2). When smoking duration was categorised (i.e., <20, ⩾20–<30, ⩾30–<40, and ⩾40 years), a positive and statistically significant trend was observed after stove improvement, whereas no trend was observed before stove improvement (HR_interaction_=1.00, 1.26, 1.51, and 1.62, *P*_trend_=0.005).

Smoking type-specific analyses showed a significant increase in pre- to post-improvement effects only for cigarette smoking, specifically unfiltered cigarettes (Table 2).

## Discussion

Cigarette smoke contains a variety of carcinogens such as PAHs, *N*-nitrosamines, and aromatic heterocyclic amines, which undergo metabolic activation by cytochrome P450s (CYPs). Major components of particulates from smoky coal combustion in Xuanwei include high levels of PAHs and methylated PAHs (Mumford *et al*, 1987, 1990). The important difference between the two exposures (i.e., smoke from cigarettes and coal) seems related to the intensity and total dose of carcinogens such as PAHs (Casale *et al*, 2001). Given that unvented Xuanwei coal smoke contains large amounts of PAHs, perhaps enough to saturate relevant enzyme systems (Lewtas *et al*, 1997) and to increase DNA repair capacity (Wei *et al*, 2000), smoking could conceivably have only a weak additional effect on lung cancer risk before stove improvement.

Another possible hypothesis for the weak effect of smoking before stove improvement may be related to activity of enzymes which activate tobacco-specific carcinogens. For example, the activity of CYP2B1, one of the major isozymes, which activate tobacco-specific NNK (4-methylnitrosamino-1-(3-pyridyl)-1-butanone), is lowered after exposure to diesel exhaust particles (DEPs) (Rengasamy *et al*, 2003) or asphalt fumes (Ma *et al*, 2003). As PAHs are a major component of smoky coal emissions, as with DEP and asphalt fumes, it is possible that very high exposure to smoky coal emissions may actually lead to lower CYP2B1 activity and therefore weakened effects of smoking on lung cancer mortality. Given insufficient experimental evidence, this hypothesis needs to be investigated by further studies.

Limitations of this study include a lack of information on change of behaviour related to ventilation after the installation of chimneys, as well as no information on clinical characteristics of the cancers occurring before and after stove improvement. Thus, we note that potential confounding cannot be excluded and our results should be interpreted cautiously. Although several previous studies have examined health outcomes related to installation of chimneys (Díaz *et al*, 2007; McCracken *et al*, 2007), our study is the first to evaluate differential effects of smoking before and after stove improvement. Our results support additional intervention to promote smoking prevention and cessation after successful reduction of indoor air pollution in this population.

In conclusion, our results suggest that the effect of smoking on lung cancer risk becomes considerably stronger after chimney installation and consequent reduction of indoor coal smoke exposure. Unvented coal smoke exposure could attenuate the effect of smoking on lung cancer mortality, especially in China where relative risks of smoking for overall mortality and lung cancer mortality are considerably lower than those to that in the west (Gu *et al*, 2009).

## Figures and Tables

**Table 1:** Selected characteristics of the subjects by stove use history in Xuanwei, 1976–1996: *n* (%) or means (95% CI)

	**Change to stove with chimney**		
	**Yes (*n*=12 529)**	**No (*n*=9127)**	**Total (*n*=21 701)**
*Person-years*	242 154	159 831	401 985
Age on 1 January, 1976, years	38.8 (38.6–39.0)	40.4 (40.2–40.6)	39.5 (39.3–39.6)
Age at exit from follow-up, years	58.1 (57.9–58.3)	57.8 (57.6–58.0)	58.0 (57.9–58.1)
			
*Lung cancer deaths, 1976–1996*	706 (5.6)	527 (5.7)	1233 (5.7)
Age at lung cancer death	56.7 (56.1–57.3)	53.9 (53.2–54.7)	55.5 (55.0–56.0)
			
People who ever used smoky coal	11 319 (90.3)	3795 (41.4)	15 114 (69.6)
Tons per year	3.44 (3.42–3.46)	2.88 (2.84–2.91)	3.30 (3.28–3.31)
			
*Age at stove improvement*	41.8 (41.6–42.0)		
Years from stove improvement to exit from follow-up	16.3 (16.2–16.4)		
			
*Ever smoked*	11 658 (93.0)	8352 (91.1)	20 010 (92.2)
Age started smoking	21.5 (21.4–21.5)	20.7 (20.6–20.8)	21.1 (21.1–21.2)
Years of smoking among smokers	33.3 (33.2–33.5)	34.3 (34.1–34.5)	33.8 (33.6–33.9)
Ever quit smoking	173 (1.4)	89 (1.0)	262 (1.2)

Abbreviation: CI=confidence interval.

**Table 2:** Effects of smoking on lung cancer mortality before and after stove improvement in Xuanwei, 1976–1996

			**Before stove improvement**		**After stove improvement**			
**Smoking variables**	***n*** a	**Lung cancer deaths**	**HR_pre_ (95% CI)** c	***P***	**HR_post_ (95% CI)** d	***P***	**HR_interaction_** b	***P*_interaction_**
*Duration of smoking of any type*								
*Duration (per year)*	21 686		0.995 (0.987–1.002)	0.17	1.009 (1.002–1.017)	0.02	**1.015**	**0.002**
<20	2949	188	1.00 (Ref.)		1.00 (Ref.)		1.00	
⩾20–<30	6971	270	0.81 (0.61–1.07)	0.14	1.02 (0.77–1.37)	0.88	1.26	0.23
⩾30–<40	5906	434	0.92 (0.69–1.22)	0.57	1.39 (1.05–1.86)	0.03	**1.51**	**0.02**
⩾40	5860	341	1.05 (0.75–1.46)	0.79	1.70 (1.23–2.34)	0.001	**1.62**	**0.01**
*P*_trend_				0.85		0.0008		**0.005**
								
*Smoking type (pack-years)*								
*Cigarettes (per pack-year)*	6394	333	0.997 (0.985–1.009)	0.61	1.015 (1.005–1.025)	0.0004	**1.018**	**0.02**
Unfiltered	5637	292	0.990 (0.976–1.004)	0.15	1.013 (1.002–1.025)	0.03	**1.024**	**0.009**
Filtered	2018	41	1.014 (0.984–1.045)	0.37	1.019 (0.997–1.042)	0.09	1.005	0.77
Chinese long-stem pipe (per (20 g day^–1^)^*^year)	8075	635	0.996 (0.991–1.002)	0.20	1.000 (0.996–1.005)	0.92	1.004	0.25
Water pipe (per (20 g day^–1^)^*^year)	2092	112	1.004 (0.987–1.022)	0.61	1.007 (0.987–1.028)	0.48	1.003	0.83

Abbreviations: CI=confidence interval; HR=hazard ratio.

a Reduced data sets included exclusive smokers of each type and non-smokers.

b HR_interaction_=HR_post_/HR_pre_.

c Adjusted HR (95% CI) before stove improvement.

d Adjusted HR (95% CI) after stove improvement. Significant results are shown in bold.
